# Barriers to uptake of early infant HIV testing in Zambia: the role of intimate partner violence and HIV status disclosure within couples

**DOI:** 10.1186/s12981-017-0142-2

**Published:** 2017-03-21

**Authors:** Karen M. Hampanda, Abigail M. Nimz, Lisa L. Abuogi

**Affiliations:** 1 Department of Community and Behavioral Health, Colorado School of Public Health, 13199 East Montview Boulevard, Suite 310, Mail Stop A090, Aurora, CO 80045 USA; 2 University of Colorado School of Medicine, 13001 E 17th Place, Aurora, CO 80045 USA; 3 Department of Pediatrics, University of Colorado School of Medicine and Children’s Hospital Colorado, Aurora, CO USA; 4 Center for Global Health, University of Colorado School of Medicine School of Public Health, 13199 East Montview Boulevard, Suite 310, Mail Stop A090, Aurora, CO 80045 USA

## Abstract

**Background:**

Early detection of pediatric HIV through uptake of infant HIV testing is critical for access to treatment and child survival. While structural barriers have been well described, a greater understanding of social and behavioral factors that may relate to maternal uptake of early infant HIV testing services is urgently needed. The aim of this study was to explore how gender power dynamics within couples affect HIV-positive women’s uptake of early infant HIV testing at a large health center in Lusaka, Zambia.

**Methods:**

In 2014, 320 HIV-positive married postpartum women were recruited at a large public health facility in Lusaka to participate in a cross-sectional survey. Data on uptake of early infant HIV testing by 4–6 weeks of age was collected through medical records. Simple and multiple logistic regression models determined significant predictors of maternal uptake of early infant HIV testing.

**Results:**

In the adjusted model, uptake of early infant HIV testing was associated with female-directed emotional intimate partner violence (aOR 0.41; 95% CI 0.21–0.79; p < 0.01), HIV status disclosure to the male partner (aOR 13.73, 95% CI 3.59–52.49, p < 0.001), and maternal postpartum ART adherence (aOR 2.28, 95% CI 1.15–4.55, p < 0.05).

**Conclusions:**

Domestic relationship dynamics, including emotional violence and HIV status disclosure to the male partner, may play an important role in maternal uptake of early infant HIV testing. These findings provide additional evidence for the link between intimate partner violence against women and poor HIV-related health outcomes. Programs that adequately screen for and address various forms of intimate partner violence within the context of prevention of mother-to-child transmission are recommended.

## Background

Despite commendable advances in prevention of mother-to-child transmission (PMTCT), pediatric HIV remains a significant public health challenge, particularly in sub-Saharan Africa. In 2015, 150,000 children under 15 years of age were newly infected with HIV and 1.8 million children were living with the virus—the majority residing in sub-Saharan Africa [1]. In the study setting, Zambia, an estimated 85,000 children were living with HIV in 2015 [2]. Early detection of HIV in infants through timely HIV testing is critical for their survival due to the increased risk of mortality without antiretroviral therapy (ART) compared to HIV-infected adults [3–5]. The World Health Organization (WHO) currently recommends testing of HIV-exposed infants in resource-limited settings using dry blood spot (DBS) polymerase chain reaction (PCR) technology at 4–6 weeks of life to optimize detection of intrauterine, intrapartum, and early postnatal mother-to-child HIV transmission [6]. Yet, most recent estimates indicate that less than half of the infants born to HIV-positive mothers in low- and middle-income countries receive an HIV test within the first two months of life [7].

Numerous healthcare system barriers to early diagnosis of HIV infection in infants in resource-limited settings have been identified. For example, DNA-PCR using DBS requires sophisticated and expensive nucleic acid testing, as well as highly trained staff and advanced laboratory infrastructure, which is generally only present in large, centralized laboratories [8–10]. Indeed, in Zambia, there are only three facilities with laboratories capable of processing PCR [11]. The situation is also exacerbated by restriction of sample collection sites to PMTCT clinics, stock outs of HIV testing commodities, poorly functioning sample transport networks, and limitations in supply chain management [8, 12, 13]. Current efforts are underway to address these acknowledged problems, including, for example, point-of-care technologies [12].

While healthcare system barriers related to infant HIV testing have been well described, less is known about the impact of social factors, including dynamics with the male partner, among HIV-positive mothers. The family—particularly the male partner—is arguably the most influential social unit affecting individual health behaviors [14]. In African settings in particular, male partners are known to play an essential role in household decision-making, including women’s use of HIV-related services [15–17]. Moreover, dynamics with male partners, such as HIV status disclosure and fear of intimate partner violence (IPV), are known to effect women’s uptake of some services in the PMTCT cascade of care [18, 19]. It is plausible that the same barriers related to dynamics within couples that exist for other PMTCT-related health behaviors may prevent HIV-positive mothers from accessing early infant HIV testing services. This study explores how gender power dynamics within couples, such as IPV against women and male controlling behaviors, women’s participation in household decision-making, and women’s relative earnings, as well as HIV status disclosure to male partners, affect HIV-positive mother’s uptake of early infant HIV testing by the recommended 4–6 weeks postpartum in a large public health center in Lusaka, Zambia.

## Methods

### Study design

A cross-sectional survey was conducted with postpartum HIV-positive women who were currently married or cohabiting with a male partner. Recruitment occurred at a large public health center during routine pediatric immunizations from April to August of 2014. Quantitative data were collected through medical records (i.e., the child’s Under-Five Card) and a face-to-face survey with closed-ended questions, including dynamics within the woman’s current heterosexual relationship. The questionnaire was pretested during a pilot study in March of 2014. Trained Zambian research assistants verbally administered the survey in the local languages on paper forms in a private location at the health center after women completed their health care visit. The questionnaire was written in English, but translated into the two most commonly spoken dialects in Lusaka (Nynaja and Bemba). Participants received a small travel reimbursement for their time. Written informed consent or a thumbprint was obtained from all study participants. The study was designed and implemented in accordance with the WHO Ethical and Safety Recommendations for Research on Domestic Violence Against Women [20]. Research Assistants were trained in research ethics. Participants who reported IPV were offered referrals to a local organization for counseling and victim support services.

### Study participants

Participants included 320 HIV-positive mothers who had brought their child for routine pediatric immunizations in the Maternal and Child Health Department of a large public health center in Lusaka, Zambia. The health center has a catchment population of over 160,000 individuals living in the surrounding low socioeconomic neighborhoods. Mothers who were diagnosed as HIV seropositive, over the age of 18 years, currently married or living with a man as if married, and whose youngest biological infant was between 3 and 9 months of age were invited to participate in the survey after completing their health care visit. Infant age criterion was selected based on the immunization schedule in Zambia and to limit recall bias. Immunization compliance in Zambia is high with 97% of all infants receiving at least some immunizations by 12 months of life [15–17].

Nurses determined eligibility for the study using the infant’s “Under-Five Card,” a mother’s copy of her child’s health record that she is required to bring to all pediatric health care visits and includes the child’s birth date, height and weight, immunizations, medications, and PMTCT care. Nurses providing immunizations examined the Under-Five Card or other medical records if the card was not available and verbally invited eligible women to participate in the survey by proceeding to a designated private room (or waiting area outside if the room was occupied) in the clinic where research assistants conducted informed consent, asked additional screening questions, and verbally administered the survey questionnaire to consenting eligible participants. The response rate for eligible women was 85%.

### Measures

The main outcome of interest, uptake of infant HIV testing by 4–6 weeks postpartum, was captured using the child’s Under-Five Card or other available medical record. Research assistants indicated on the survey questionnaire whether the child had record of HIV testing at 4–6 weeks via PCR, and if available, the result of the test. Gender power dynamics within the couple, the independent variables of interest, were measured using the Revised Conflict Tactics Scale (CTS2). The CTS2 is one of the most widely used gender power measurement tools worldwide with strong reported psychometric properties [21]. The version of the CTS2 used in this study came directly from the Zambian Demographic and Health Survey (ZDHS) Domestic Violence Module [22, 23]. The DHS Domestic Violence Module was first developed and standardized in 2000 and has been continually used throughout sub-Saharan Africa [24]. The module includes questions regarding different forms of IPV against women, male partner controlling behaviors, women’s participation in household decisions, and women’s relative earnings. Participants were asked to report their experiences with specific emotional, physical, and sexual IPV events ever occurring in the course of their relationship with the current male partner. There were three possible specific events for emotional violence, seven possible events for physical violence, and two possible events for sexual violence. Participants also reported whether their male partner displays specific controlling behaviors from a set of six questions. In addition, the survey included four questions regarding women’s participation in household decisions regarding daily household purchases, major household purchases, women’s use of health care, and the final say over how money is spent. Women’s relative earnings compared to the husband was measured by asking women if the they earn more money than the husband, about the same amount of money, or less money than the husband. We also added one additional question on women’s HIV status disclosure to the husband.

Control variables included the woman’s age, infant’s age, parity, education, household wealth (a proxy for socioeconomic status), when the woman was diagnosed with HIV (before or during the most recent pregnancy), if the woman was taking lifelong triple ART or short course prophylaxis under WHO Option A, knowledge of PMTCT, the woman’s postpartum ART adherence, and the male partner’s HIV status (positive, negative, or unknown). Educational attainment was measured by asking women “what was the highest level of school you attended” (none, some primary, completed primary, some secondary, completed secondary, some college, completed college, or graduate level). We measured socioeconomic status by asking participants what household assets their home owned from a list of 21 possible items, including, for example, electricity, a car, and a refrigerator. Knowledge of PMTCT was measured by asking participants “are there any special drugs that a doctor or nurse can give to a woman infected with HIV to reduce the risk of transmission to the baby?.” Lastly, postpartum ART adherence was measured using a visual analog scale (VAS) [25] that was verbally explained to women by the research assistants. Participants retrospectively reported on their estimate of ART adherence (doses taken at the appropriate time) since giving birth from 0 to 100%. Postpartum adherence rather than pregnancy adherence was included in our analysis because the time period more closely correlates with maternal uptake of infant HIV testing behaviors.

### Data cleaning and statistical analysis

Survey data were double entered into CSPro and exported into Stata 12 for analysis. Surveys with more than 50% missing data were not included in the analyses (n = 4). Missing data (2.3%) were imputed using multivariate chained equations in Stata 12 [26, 27]. Data converged, indicating that the multivariate chained model was a good fit for the data set [28]. We created a binary dependent variable for uptake of early infant HIV testing based on whether or not a PCR test was specified on the child’s health record/Under-Five Card by 4–6 weeks of age. For the independent variables regarding dynamics with the male partner, we created two binary variables to capture experiences with IPV: 1) if the woman reported any female-directed emotional IPV in the course of the relationship and 2) if the woman reported any female-directed physical/sexual IPV in the course of the relationship. We also created a binary variable to capture if the male partner displayed three or more controlling behaviors based on prior literature standards for analyzing this variable [24]. Additionally, we created a count variable to indicate the number of household decisions (0–4) women reported participating based on their responses to the individual household decision-making questions. Women were categorized as participating in a household decision if they made the decision alone or together with the male partner, compared to when the male partner alone makes the decision. For women’s relative earnings, we created a binary variable to indicate whether women make more/about the same amount of money as the husband, compared to women who make less money than the husband. Lastly, a binary variable for status disclosure was created to capture whether women reported notifying their male partner of their HIV-positive status.

For the control variables, the woman’s age and parity were treated as continuous variables in the analysis. Two variables based on reported educational attainment were created: if the woman completed primary education and if the woman completed secondary education. For socioeconomic status, we converted women’s responses to the list of household assets into a linear index using principal-components analysis (PCA) to derive weights [29]. For example, if a household had electricity, it would move the participant 0.30 on the index; however, if a household owned a chair, it would only move the participant 0.17 on the index. The index was standardized prior to inclusion in the regression models. Additional binary control variables included: whether the woman was diagnosed with HIV during the most recent pregnancy, whether she was taking lifelong triple ART, and whether she had knowledge regarding PMTCT. Postpartum ART adherence was dichotomized into a variable indicating if the woman was at least 80% adherent to taking medication after giving birth based on recent studies suggesting this is the adequate doses needed to suppress the virus [30, 31]. Finally, three dummy variables were created to indicate the male partner’s HIV status: (1) if the male partner was HIV-positive, (2) if the male partner had an unknown status or never tested for HIV, and (3) if the male partner was HIV-negative.

Descriptive statistics highlighted sample characteristics stratified by if the child had an early HIV test. Simple logistic regression models determined significant difference in the odds of early infant HIV testing by the independent variables of interest and control variables. We also ran a pairwise correlation to determine the correlation coefficients for experiencing any emotional IPV in the course of the relationship, the number of household decisions the woman participates, and if the male partner displays 3 or more controlling behaviors. These gender power variables were selected for the correlation analysis because they were significantly associated with early infant HIV testing in the bivariate model. Each measure in the correlation analysis was highly correlated with the other (p < 0.001), indicating a significant interaction between these variables.

Finally, a multiple logistic regression model determined significant differences in the odds of early infant HIV testing by the relevant independent variables (i.e., those associated with the dependent variable in the simple logistic regression models) after adjusting for control variables of age, parity, whether the woman completed secondary education, whether she was diagnosed with HIV during the most recent pregnancy, household wealth index, postpartum ART adherence, and the husband’s HIV status. Secondary education, rather than primary education, was included in the multiple logistic regression models because it displayed greater variability in the sample. The variables capturing knowledge of PMTCT, if the woman was taking lifelong triple ART, any female-directed physical/sexual IPV, and if the woman had greater or equal earnings as male partner were not included in the multiple logistic regression model because they were not significantly associated with early infant HIV testing in the simple logistic regression models and have not previously been established in the literature as important covariates.

## Results

### Participant characteristics

Table 1 displays the participant characteristics, comparing infants who had an early HIV test and those who did not. Of the 320 HIV-positive female participants, the median age was 28 years (IQR 24, 34). Women had given birth to a median of 3 children (IQR 2, 4). Over 70% of women had completed primary education, but only 14% had completed secondary education. For the poorest 40% of the sample, the mean wealth index score was −1.97. For the mid-wealth participants (also 40% of the sample), the mean wealth index score was 0.88. For the wealthiest 20% of the sample, the mean wealth index score was 2.67. In terms of actual household assets, 75% of participants had electricity; 39% owned a refrigerator, and 5% owned a vehicle. Knowledge of PMTCT was nearly universal: 96% of women agreed that there are special drugs that can prevent mother-to-child HIV infection. The majority of participants (60%) reported being diagnosed with HIV during the most recent pregnancy while 40% knew their status prior to becoming pregnant. Fifty-six percent of participants were prescribed lifelong triple ART while 44% were not eligible for treatment and taking short-course prophylaxis under WHO programmatic Option A. Lastly, 81% of women reported being adherent to ART postpartum.

**Table 1:** Participant Characteristics (n = 320)

Variable	Infant tested by 4–6 weeks (n = 233)	Infant not tested by 4–6 weeks (n = 87)	Total (n = 320)	
	Median (IQR)/N (%)	Median (IQR)/N (%)	Median (IQR)/N (%)	P
Demographic characteristics				
Age in years	29.0 (25.0, 34.0)	27.0 (24.0, 33.0)	28.0 (24.0, 34.0)	0.097
Parity	3.0 (2.0, 5.0)	3.0 (2.0, 4.0)	3.0 (2.0, 4.0)	0.161
Completed primary education	167 (72.8%)	63 (72.2%)	230 (71.9%)	0.572
Completed secondary education	35 (15.3%)	9 (11.4%)	44 (13.8%)	0.187
Standardized wealth Index	0.14 (−0.73, 0.89)	−0.08 (−0.83, 0.59)	0.06 (−0.78, 0.76)	0.106
Diagnosed with HIV during most recent pregnancy	130 (55.9%)	63 (72.0%)	192 (60.3%)	0.010
Prescribed lifelong triple ART^a^	138 (59.1%)	41 (47.7%)	179 (56.0%)	0.068
Knowledge of PMTCT	225 (96.5%)	81 (93.0%)	306 (95.6%)	0.183
Adherent to ART^b^	202 (86.8%)	57 (65.1%)	259 (80.9%)	0.000
Sexual relationship characteristics				
Disclosed HIV status to male partner	227 (97.6%)	66 (76.0%)	293 (91.8%)	0.000
Male partner HIV status				
Positive (seroconcordant)	128 (55.2%)	36 (41.9%)	164 (51.6%)	0.036
Negative (serodiscordant)	65 (27.8%)	24 (27.6%)	89 (27.8%)	0.980
Unknown/partner not tested	40 (17.0%)	26 (30.5%)	66 (20.6%)	0.010
Any female-directed emotional IPV in course of relationship	79 (33.9%)	50 (57.9%)	129 (40.3%)	0.000
Any female-directed physical/sexual IPV in course of relationship	113 (48.5%)	47 (54.4%)	160 (50.0%)	0.356
Male partner displays 3 + controlling behaviors^c^	131 (56.4%)	63 (72.0%)	194 (60.6%)	0.012
Number of household decisions the woman participates^d^	3.0 (2.0, 4.0)	2.0 (1.0, 3.0)	2.0 (1.0, 4.0)	0.007
Woman has greater or equal earnings as male partner	59 (25.3%)	17 (19.7%)	76 (23.8%)	0.294

^a^Comparison group: prescribed short-course prophylaxis under Option A

^b^Defined as the woman’s self-report of taking >80% of medication doses postpartum (includes both short-course prophylaxis and lifelong triple ART)

^c^Out of six total behaviors

^d^Out of four total household decisions

Disclosure of women’s HIV status to the male partner was high with 92% of women reporting they had informed the current partner of their HIV-positive status. Approximately half (52%) of the participants’ reported having a male partner who is also living with HIV (i.e., seroconcordant), 28% reported having a male partner who is HIV-negative (serodiscordant), and 21% reported having a male partner whose status was unknown or who had never tested for HIV. Experiences with IPV were common: 40% of women reported any female-directed emotional IPV in the course of the relationship and 50% of women reported any female-directed physical/sexual IPV in the course of the relationship. Male controlling behaviors were also common with 56% of women reporting their male partner displays three or more controlling behaviors. Women reported participating in a median of half (2.0 out of 4.0) of the household decisions. Twenty-five percent of women reported having equal or greater earnings as the male partner.

### Uptake of early infant HIV testing and unadjusted associations

Seventy-three percent (n = 233) of infants had an HIV test by 4–6 weeks of age. Table 1 indicates which demographic and sexual relationship characteristics were associated with uptake of early infant HIV testing in the simple logistic regression models. Two demographic characteristics were associated with uptake of early infant HIV testing in the bivariate models: knowing one’s HIV status prior to becoming pregnant (p < 0.05) and being adherent to postpartum ART (p < 0.001). Sexual relationship characteristics associated with uptake of early infant HIV testing included: HIV status disclosure to the male partner (p < 0.001), having an HIV-positive male partner (p < 0.05) and knowing the male partner’s HIV status (p < 0.05), not experiencing any female-directed emotional IPV (p < 0.001), the male partner displaying <3 controlling behaviors (p < 0.05), and greater participation in household decision-making (p < 0.01).

### Uptake of early infant HIV testing and adjusted associations

Table 2 displays the multiple logistic regression results for the adjusted odds of early infant HIV testing by dynamics within the couple and control variables. The only power dynamic that remained significantly associated with uptake of early infant HIV testing after including other gender power measures and control variables was female-directed emotional IPV: women who reported any female-directed emotional IPV in the course of their relationship had 59% lower adjusted odds of early infant HIV testing (aOR 0.41; 95% CI 0.21–0.79; p < 0.01), compared to women who did not experience emotional IPV. Conversely, women’s participation in household decisions was not significantly associated with early infant HIV testing, nor was the male partner displaying three or more controlling behaviors after adjusting for emotional IPV and other control variables. Women who reported disclosing their HIV status to the male partner, however, had 14 times higher adjusted odds of early infant HIV testing (aOR 13.73, 95% CI 3.59–52.49, p < 0.001), compared to women who did not disclosure their status. Finally, women who reported >80% adherence to ART after birth had 2 times higher adjusted odds of early infant HIV testing (aOR 2.28, 95% CI 1.15–4.55, p < 0.05), compared to women who reported <80% ART adherence and after adjusting for dynamics with the male partner and other control variables.

**Table 2:** Multiple logistic regression results for the adjusted odds of early infant HIV testing

	Infant HIV testing by 4–6 weeks aOR (95% CI)
Dynamics with the male partner	
Any female-directed emotional IPV in course of relationship	0.41** (0.21–0.79)
Number of household decisions the woman participates	1.10 (0.86–1.39)
Male partner displays 3 + controlling behaviors	1.05 (0.53–2.06)
Disclosed HIV status to male partner	13.73*** (3.59–52.49)
Control variables	
Age in years	1.03 (0.96–1.11)
Parity	1.03 (0.80–1.32)
Completed secondary education	2.09 (0.81–5.40)
Standardized wealth Index	1.04 (0.76–1.40)
Diagnosed with HIV during most recent pregnancy	0.66 (0.36–1.22)
Maternal ART adherence postpartum	2.28* (1.15–4.55)
Male partner HIV status:	
HIV-Negative	ref.
Unknown	1.68 (0.67–4.19)
HIV-positive	1.13 (0.58–2.19)

* p < 0.05; ** p < 0.01;*** p < 0.001

## Discussion

Over one-quarter of the HIV-exposed infants in this study failed to receive early PCR testing by the recommended 4–6 weeks—hindering early diagnosis and treatment, and the odds of survival [3–5]. This study demonstrates that dynamics with the male partner may have a significant affect on the odds of maternal uptake of early infant HIV testing. In this study, female-directed emotional IPV was strongly associated with reduced adjusted odds of early infant HIV testing. Notably, there was a significant interaction between female-directed emotional IPV, women’s participation in household decisions, and male partner controlling behaviors—with emotional IPV driving the relationship with uptake of infant HIV testing. Our findings add to the growing body of literature linking different forms of IPV to poor medical outcomes across the continuum of care for HIV-infected mothers and their children [19, 32–34]. Our findings also underscore the importance of examining IPV as a multidimensional phenomenon [35] and including emotional/psychological forms of abuse as potential predictors for poor health outcomes.

We hypothesize several potential explanations for the relationship between emotional IPV and reduced odds of early infant HIV testing, which warrant further exploration. First, partners in emotionally violent relationships may have poor communication, which is known to be important for other PMTCT-related health behaviors [36–38]. In addition, male partners who are emotionally violent may be less willing to participate in PMTCT-related care, which, again, is a known facilitator for uptake of other PMTCT protocols [39–41]. Lastly, women experiencing emotional IPV may be afraid of finding out the infant is HIV-positive and enduring additional potential emotional abuse from their male partner. We recommend that future qualitative research with peripartum HIV-positive women explore the mechanisms within emotionally violent relationships that are different than those in physically/sexually violent relationships, which may impede the uptake of HIV-related health behaviors differentially.

Another dynamic within sexual relationships related to early infant HIV testing is women’s HIV status disclosure to male partners. This study supports prior research from sub-Saharan Africa indicating women’s HIV status disclose to male partners improves participation in PMTCT programs [42, 43]. In this study, we document a direct quantitative relationship between non-disclosure of women’s HIV status to male partners and lower uptake of early infant HIV testing. Recent qualitative work also indicates that women’s fear of stigma and involuntary HIV status disclosure to family members or the community are barriers to early infant diagnosis and the continuation of HIV care for mothers and their children [44, 45].

Outside of dynamics with the male partner, maternal HIV-related health behaviors also affect the odds of early infant HIV testing. Our findings indicate that women who reported better postpartum ART adherence were more likely to have their child tested for HIV by 4–6 weeks. Importantly, this finding suggests that the HIV-exposed children who are at the greatest risk of contracting HIV (due to maternal non-adherence to PMTCT) may be least likely to be diagnosed through early infant HIV testing. It is therefore critical for PMTCT programs to promote maternal adherence early in the cascade of care and prioritize outreach and counseling with women who are non-adherent to ART—both for their own health and to ensure that they are not lost to follow-up and miss opportunities for early infant HIV testing.

Our prior research indicates that IPV against HIV-positive women, including emotional IPV, negatively affects adherence to several steps in the PMTCT cascade, including maternal ART adherence during and after pregnancy [32]. The present study adds that early infant HIV testing, a critical step in the cascade of care for HIV-exposed infants, is also affected by emotional IPV. Thus, programs within the context of PMTCT that adequately screen for and address various forms of IPV are urgently needed. Counseling within PMTCT programs should attempt to evaluate women’s home circumstances and determine who may be at risk for low compliance because of IPV, including emotional abuse. An appropriate referral system between PMTCT programs and IPV services should also be prioritized.

The results of this study should be interpreted within several limitations. First, it is cross-sectional and cannot establish causality or the timing of events. Second, status disclosure and gender power dynamics are based on self-report, which can be vulnerable to recall and social desirability biases. The sample is also non-representative and clinic-based, limiting the generalizability of findings. This study may also have selection bias because all participants were bringing their children for pediatric immunizations, which may not be representative of all mother-baby pairs needing infant HIV testing. We did not measure communication within the couple or male partner support for health behaviors, which may be mediating factors in the relationship between emotional IPV and uptake of early infant HIV testing. We were also unable to test the relationship between dynamics within couples and the odds of the infant being diagnosed with HIV because only one-third of children had any available test results due to the slow turn-around time for PCR in Zambia at the time of the study.

## Conclusion

This study highlights the role of dynamics within heterosexual couples and uptake of early infant HIV testing. Emotional IPV and non-disclosure of an HIV-positive status to male partners are strongly associated with reduced odds of early infant HIV testing. Given the high prevalence of IPV in the region, including emotional IPV, these findings have far-reaching implications for early infant HIV diagnosis and treatment. Programs that adequately screen for and address various forms of IPV within the context of PMTCT are recommended.
